# The Intra- and Inter-Rater Reliability of an Instrumented Spasticity Assessment in Children with Cerebral Palsy

**DOI:** 10.1371/journal.pone.0131011

**Published:** 2015-07-02

**Authors:** Simon-Henri Schless, Kaat Desloovere, Erwin Aertbeliën, Guy Molenaers, Catherine Huenaerts, Lynn Bar-On

**Affiliations:** 1 Clinical Motion Analysis Laboratory, University Hospital Leuven, Leuven, Belgium; 2 Department of Rehabilitation Sciences, KU Leuven, Leuven, Belgium; 3 Department of Mechanical Engineering, KU Leuven, Leuven, Belgium; 4 Departments of Development and Regeneration, KU Leuven, Leuven, Belgium; 5 Department of Orthopaedic Medicine, University Hospital Leuven, Leuven, Belgium; Duke University, UNITED STATES

## Abstract

**Aim:**

Despite the impact of spasticity, there is a lack of objective, clinically reliable and valid tools for its assessment. This study aims to evaluate the reliability of various performance- and spasticity-related parameters collected with a manually controlled instrumented spasticity assessment in four lower limb muscles in children with cerebral palsy (CP).

**Method:**

The lateral gastrocnemius, medial hamstrings, rectus femoris and hip adductors of 12 children with spastic CP (12.8 years, ±4.13 years, bilateral/unilateral involvement n=7/5) were passively stretched in the sagittal plane at incremental velocities. Muscle activity, joint motion, and torque were synchronously recorded using electromyography, inertial sensors, and a force/torque load-cell. Reliability was assessed on three levels: (1) intra- and (2) inter-rater within session, and (3) intra-rater between session.

**Results:**

Parameters were found to be reliable in all three analyses, with 90% containing intra-class correlation coefficients >0.6, and 70% of standard error of measurement values <20% of the mean values. The most reliable analysis was intra-rater within session, followed by intra-rater between session, and then inter-rater within session. The Adds evaluation had a slightly lower level of reliability than that of the other muscles.

**Conclusions:**

Limited intrinsic/extrinsic errors were introduced by repeated stretch repetitions. The parameters were more reliable when the same rater, rather than different raters performed the evaluation. Standardisation and training should be further improved to reduce extrinsic error when different raters perform the measurement. Errors were also muscle specific, or related to the measurement set-up. They need to be accounted for, in particular when assessing pre-post interventions or longitudinal follow-up. The parameters of the instrumented spasticity assessment demonstrate a wide range of applications for both research and clinical environments in the quantification of spasticity.

## Introduction

Cerebral Palsy (CP) is the most common neurological disorder in children. It is the result of an upper motor neuron (UMN) lesion in the immature brain. Spasticity is identified in 80–90% of children with CP [1]. Excessive and/or unmanaged spasticity causes pain, limits functional ability, and contributes to secondary complications such as muscle contracture and bone deformity [2]. Despite the detriment of spasticity, there exist only a handful of clinically feasible assessments. Ambiguity over a precise definition of spasticity [3] may be central to this shortcoming.

Perhaps the most commonly cited definition refers to ‘a velocity-dependent increase in tonic stretch reflex with exaggerated tendon jerks, resulting from hyper-excitability’ [4]. Another common citation also incorporates the resistance felt due to an externally imposed movement, increasing with speed of stretch, or above a threshold speed or joint angle [5]. Non-neural related muscle and tendon stiffness also contribute to this resistance, especially in persons with an UMN syndrome [6]. Distinguishing the resistance due to a hyperactive stretch reflex from an increased passive stiffness is clinically very challenging.

In clinical environments, spasticity is routinely measured by means of subjective, easy to use, time-efficient manual clinical scores, grading the level of resistance felt by the assessor during a passive muscle stretch. The Modified Ashworth Scale (MAS) [7] and the Modified Tardieu Scale (MTS) [8] are the most common examples. Despite their frequency of use, both have been criticized for their oversimplification of spasticity evaluation [9]. Several studies have shown that MAS and MTS are incapable of differentiating between neural and non-neural contributions to increased resistance [10]. Furthermore, various studies have highlighted the subjective nature of these assessments, which leads to poor intra- and inter-rater reliability, especially when assessing the muscles of the lower limb, as opposed to the muscles of the upper limb [6,11,12].

This necessitates the need for an objective, quantitative, robust measurement tool, feasible for the clinical environment. It is arguably indispensable for the accurate evaluation of spasticity, and for providing the correct and appropriate course of treatment [10,11].

An instrumented biomechanical approach provides a more quantitative evaluation of resistance when compared to manual clinical scores. For example, motor-driven isokinetic devices displace a limb at a controlled velocity, measuring limb resistance to passive movement [13,14]. Using surface electromyography (sEMG) investigates a muscle’s electrical activity in response to passive or active movements [15,16]. Fewer studies have simultaneously interpreted muscle activity with resistance and velocity measurements. Such an integrated approach is ideal as it considers both the neurophysiological and biomechanical methods [10,11], and assists in differentiating the components of increased resistance. This may help identify why some children respond more positively to spasticity treatment, and ensures that a child with CP receives therapy tailored to the mechanisms contributing to his or her specific symptoms.

However, combining these recommendations requires some compromise. A new method should be more valid and reliable than the current clinical scores, but remain clinically feasible in different patient pathologies and age groups. For example, motor-driven isokinetic devices measure limb resistance to passive movement with high reliability [13,14,17,18], but are often bulky and difficult to apply to children for high-velocity stretches [11]. Furthermore, a stretch reflex may be easier to excite by a transient acceleration, which is robotically more difficult to apply [19]. Therefore, a manually controlled instrumented displacement method offers a more attractive and clinically relevant alternative [20–22]. However, since spasticity is considered to be force- and velocity-dependent, the interaction between patient and examiner may affect the measurement, so a manually controlled displacement method must follow a standardized protocol, and its psychometric properties should be well defined before it is used in clinical practice [11].

Reliability is considered as the basic psychometric criterion for assessment tools. Without it, the consistency of a measurement cannot be evaluated [23], and consequently, the effect of intervention cannot be determined. Some variations arise from methodological errors, and can be considered as indications for improving the quality of the measurement (extrinsic errors), whilst other errors occur naturally, and can only be measured and accounted for (intrinsic errors) [24]. In a spasticity assessment, the variability of sequential stretch repetitions is a measure of the inherent intrinsic error. Preparation of the skin for sEMG placement, participant and limb positioning, time of day and activity prior to measurement are examples of extrinsic errors.

A manually controlled Instrumented Spasticity Assessment (ISA) was recently developed and validated to identify the severity of spasticity in the muscles of children with CP, and distinguish them from the muscle behaviour in typically developing (TD) children [25]. ISA has also been used to evaluate intervention responsiveness to botulinum toxin type-A (BTX) injections in the medial hamstrings [26]. However, until now, a comprehensive reliability study of both the intra- and inter-rater assessments, with an exploration of the influence of various sources of intrinsic and extrinsic error, has yet to be established. The current study aims to evaluate the intra-rater within session, the inter-rater within session, and the intra-rater between session reliability of various performance- and spasticity-related parameters collected with ISA in children with CP. It was hypothesised that a) the parameters assessed with ISA are overall reliable, and b) the data selection procedure does not contribute significantly as a source of extrinsic error.

## Methodology

### Participants

Twelve participants were recruited from the Clinical Motion Analysis Laboratory, University Hospital of Pellenberg. The inclusion criteria were: (1) diagnosis of spastic CP; (2) 5–18 years of age; and (3) the ability to understand and perform the test procedure. Children were excluded if they had received BTX injections six months prior to the assessment; an intrathecal baclofen pump; selective dorsal rhizotomy; or lower limb orthopaedic surgery. The Ethical Committee of the University Hospitals of Leuven approved the experimental protocol (s50808) and written informed consent for participation was acquired from all parents.

### Data acquisition

**Fig 1:**
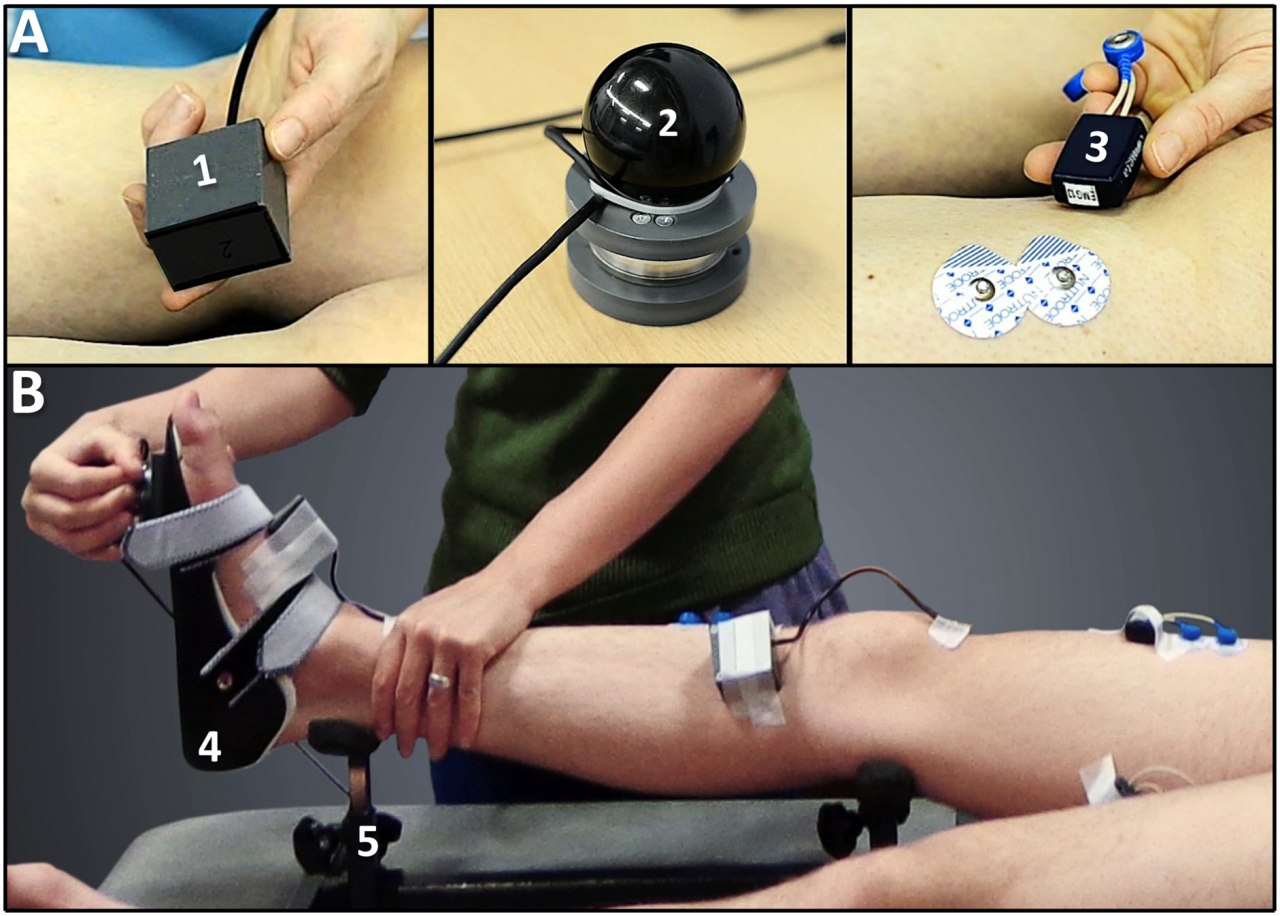
A. Measurement instrumentation. (1) three inertial measurement units (joint angle measurement); (2) a six degrees of freedom force/torque-sensor (torque measurement); (3) surface electromyography (muscle activation measurement); B. Measurement set-up for assessing the lateral gastrocnemius. (4) custom ankle orthosis; and (5) support frame. [25].

ISA has previously been reported and described [25]. The device has three components (Fig 1): (1) joint angle characteristics are measured using three inertial measurement units (IMUs: Analog Devices, ADIS16354) at a sample rate of 200 Hz; (2) reactive resistance is measured using a six degrees of freedom force/torque load-cell (ATI mini45: Industrial Automation) at a sample rate of 200 Hz; (3) sEMG activity of agonist and corresponding antagonist muscle is evaluated with a telemetric Zerowire system (Cometa, Milan, IT) at a sample rate of 2000 Hz. Labview (version 8.5, National Instruments) was used for data acquisition.

### Measurement

The four muscles evaluated with ISA were: the lateral belly of the gastrocnemius (LatGas), medial hamstrings (MedHam), rectus femoris (RecFem) and the hip adductors (Adds). These muscles were selected as they are frequently treated for spasticity [8], and are also superficial, which is necessary for acquisition with sEMG. Prior to ISA, all participants underwent a lower limb clinical assessment, including evaluation of passive range of motion (ROM), muscle strength, and muscle selectivity [25]. The MAS and MTS were performed to provide a notion of spasticity. The MAS was performed for all four muscle groups, and in addition, the MTS was performed for the gastrocnemius and hamstrings in cases where a MAS ≥1+ was given. In children with unilateral involvement, the affected side was tested. In children with bilateral involvement, the most affected side (highest average MAS-score, or, in case of symmetrical MAS-scores, the most severe MTS score) was tested. Body-weight, height and length of lower limb segments (full leg, from superior iliac spine to medial malleolus; lower-leg, from the tibia-femoral joint space to the medial malleolus; foot, from lateral malleolus to the head of metatarsal two) were recorded.

### Preparation

Preparation prior to data collection consisted of shaving and cleansing the skin, and application of the sEMG electrodes [25]. One IMU was placed on each segment (thigh, shank, and foot) in positions not interfering with the placement of the sEMG electrodes. IMU placement was arbitrary as calibration trials were carried out during the measurement (S1 Fig [25]). The force/torque loadcell was calibrated and attached to the appropriate limb segment with an orthosis. Measurements of LatGas, MedHam, and RecFem were carried out with the participant in supine lying. Measurement of the Adds was carried out in side lying. For the latter measurement, the force/torque sensor was omitted, as the leg was deemed too heavy to balance on the sensor.

### Protocol

**Fig 2:**
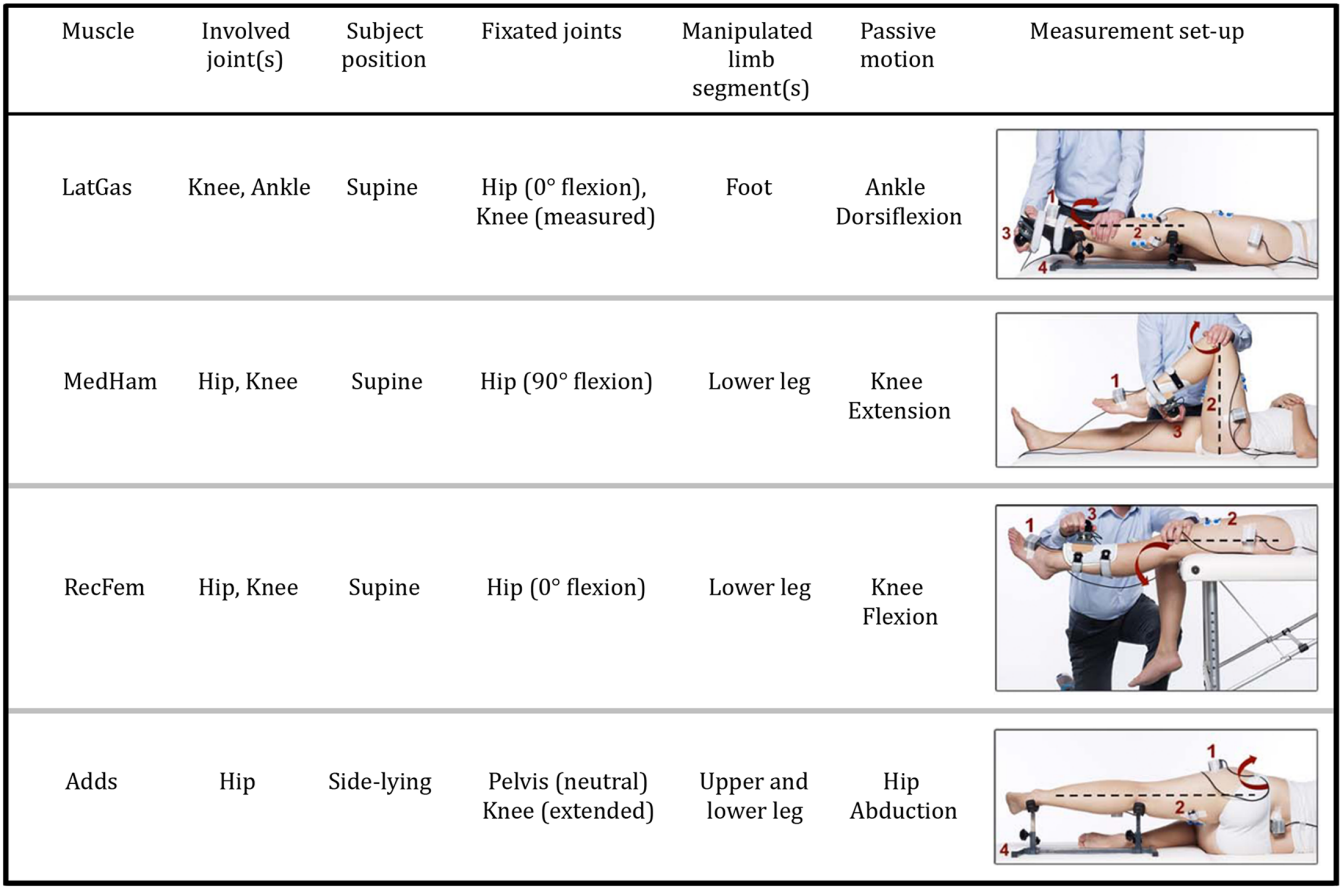
Measurement procedure for the four lower limb muscles. LatGas, lateral gastrocnemius; MedHam, medial hamstrings; RecFem, rectus femoris; Adds, hip adductors. The red arrow indicates the direction of joint movement during stretch. Instrumentation: (1) three inertial measurement units (joint angle measurement); (2) surface electromyography (muscle activation measurement); and (3) a six degrees of freedom force/torque sensor attached to a shank or foot orthosis (torque measurement); (4) support frame. Modified from [30] with permission (S2 Fig).

Data collection began with three repetitions of a maximum voluntary isometric contraction (MVIC) for each muscle. IMU calibrations for the ankle, knee and hip were performed, and moment arms were measured with a tape measure. Four repetitions of a manually applied passive muscle stretch at three incremental velocities were performed for each muscle. Low velocity (LV) corresponded to moving the hip, knee or ankle over the available ROM during five seconds, the medium velocity was an intermediate stretch of approximately one second (not included in the current data analysis) and the third, a high velocity (HV) stretch, was performed as fast as possible. The interval between stretch repetitions was seven seconds, to avoid the effects of decreased post activation depression in spastic muscles [27]. This stemmed from the five seconds [28], and 10–15 seconds [29] proposed by other groups in literature. An overview of the measurement protocol per muscle can be found in Fig 2.

### Research design

**Fig 3:**
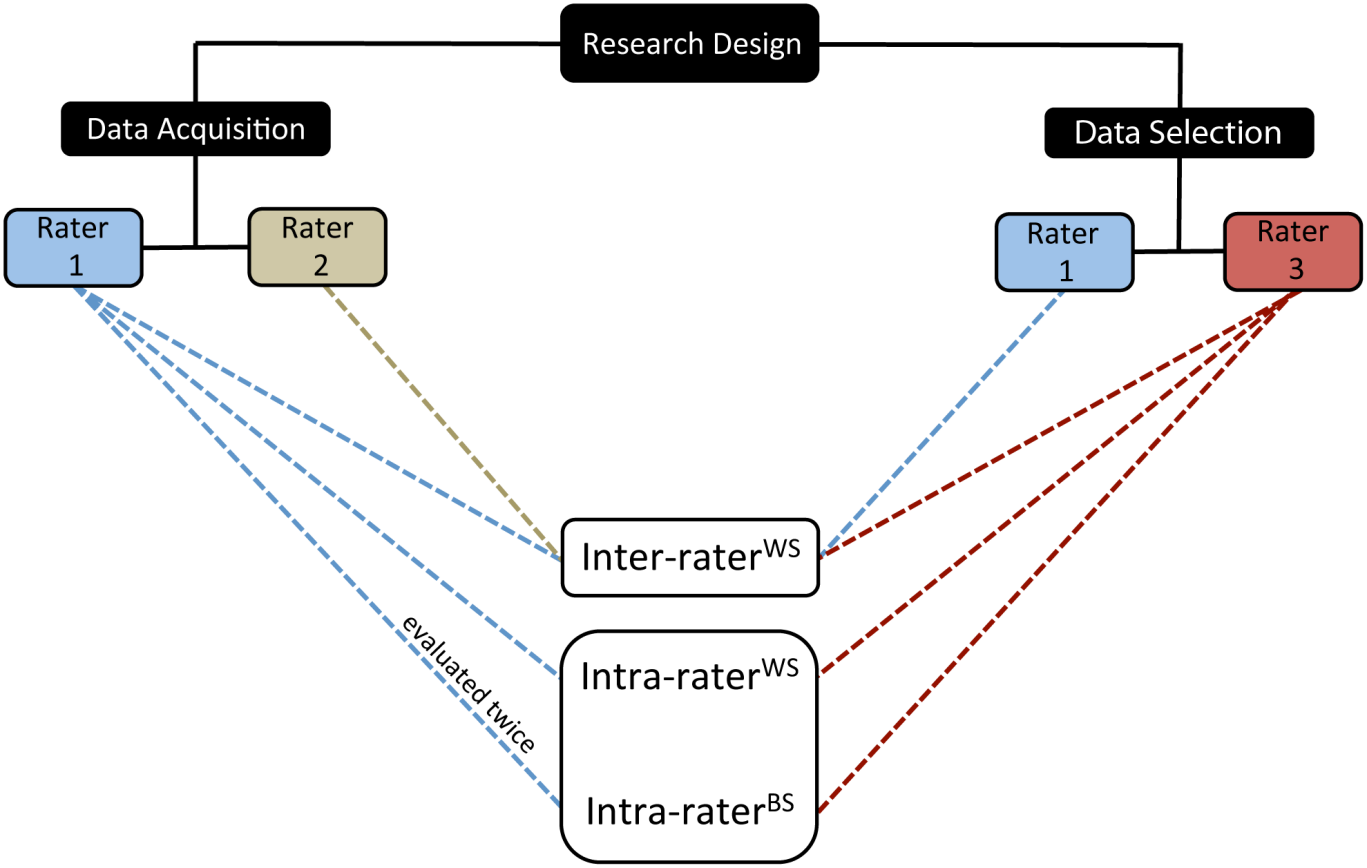
Schematic illustrating the three aspects of reliability evaluated within this study. Inter-rater^WS^, inter-rater within sessions; Intra-rater^WS^, intra-rater within sessions; Intra-rater^BS^, intra-rater between session. The dotted lines indicate the involvement of each rater in their respective analysis.

Three aspects of reliability were assessed in this study (Fig 3). Sets of stretch repetitions were performed consecutively by two trained raters in a randomised order (coin flipping), which allowed for evaluation of the inter-rater within session (inter-rater^WS^) reliability. During this analysis, the participant stayed in the evaluation room, and the sensors were not removed. Comparison between the first three good quality stretch repetitions carried out during this session by the first rater provided the data for the evaluation of the intra-rater within session (intra-rater^WS^) reliability. Upon completion, all sensors were removed and the participant was given a two-hour resting period to allow for washout, during which the participant was in the hospital cafeteria. Following the break, the first rater reapplied all the sensors, and measured the participant for a second time for the evaluation of the intra-rater between session (intra-rater^BS^) reliability. The consistency of data selection was also evaluated (see data selection section).

### Data analysis

The data from the acquired LV and HV stretches were processed in MATLAB (version 8.1.0.604 R2013a: MathWorks). The raw sEMG signal was filtered with a 6^th^ order zero-phase Butterworth bandpass filter from 20 to 500 Hz. The root mean square (rms) envelope of the sEMG signal (rms-EMG) was extracted by applying a low-pass 30Hz 6^th^ order zero-phase Butterworth filter on the squared signal. EMG onset was defined on the rms-EMG signal as the time of the first muscle activity according to the method of Staude and Wolf [31]. In cases where this method failed (i.e. no onset or constant activation), a threshold method was applied (onset = rms-EMG activity 2SD >baseline during a 0.05s interval). To estimate joint angles, a Kalman smoother [32] was applied to the data from the IMUs. Muscle lengths were estimated based on the joint angles and anthropometric data using OpenSim software [33]. The torque signals were processed with a low-pass filter with a cut-off frequency of 40Hz [21]. The net internal joint torque was calculated from the segment lengths, moment arms, exerted forces and moments, and the external forces caused by gravity and inertia [34] (see S1 Fig for a detailed overview of the different torque components).

### Data selection

For the data acquired from the three analyses, a blinded, independent third rater performed the data selection. In addition, to assess the reliability of the selection procedure, the first rater also selected the data from the inter-rater^WS^ analysis (Fig 3). Data selection was performed by visualising the raw- and processed data signals in MATLAB. Any questionable performance of a stretch repetition annotated during the acquisition was taken into account during data selection.

Reasons for excluding stretch repetitions were due to poor performance or poor quality data. Performance-related reasons for data exclusion included poor handling of the force/torque sensor (mentioned during the acquisition), inconsistent maximum stretch velocities within one trial (for LV, stretch repetitions that were >7°/s from the average of all the repetitions; for HV, stretch repetitions that were >40°/s from the average of all the repetitions, derived from previously collected data [26]), or stretches that were performed outside the desired plane of motion (forces and torques registered in directions other than the sagittal plane). Poor quality EMG included clear artefacts in the EMG signal, loss of the EMG signal, a highly inconsistent EMG pattern in comparison with the other stretch repetitions, low signal-to-noise ratio or active assistance of the participant during the passive stretches (activation of agonist and/or antagonist prior to stretch onset or at inconsistent moments during stretch). The automatic definition of EMG onset was visually inspected. In those cases when neither automatic EMG onset detection method was successful, the third rater manually determined the EMG onset based on visual inspection.

### Outcome parameters

Twelve parameters based on previous ISA literature [24,34,35] were selected and categorised as either performance-related (five parameters) or spasticity-related (seven parameters).

#### Performance-related

Performance-related parameters were used to evaluate the quality of the performance of the stretch repetitions. They included the ROM covered during LV and HV stretches (ROM^LV^ and ROM^HV^, respectively). The maximum velocity reached during LV and HV stretches (V_MAX_^LV^ and V_MAX_^HV^, respectively), and the single largest value of the rms-EMG amplitude acquired from the three MVIC repetitions (peak MVIC).

#### Spasticity-related

Spasticity-related parameters were extracted from rms-EMG and from the computed net internal joint torque. A ‘zone of maximum velocity’ (V_max_zone) was demarcated in order to emphasise the velocity-dependent character of spasticity. The V_max_zone was defined as starting 200ms prior to V_MAX_ and ending at 90% of the full ROM of the stretch. Average rms-EMG was calculated by dividing the area under the rms-EMG time curve by the duration of the V_max_zone (rms-EMG, expressed in mV). This parameter was also expressed as a normalised percentage to the peak MVIC (rms-EMG, expressed as %). Torque (expressed in Nm) was analysed at 70° knee flexion for the MedHam and RecFem, and at 10° plantar flexion for the LatGas. These angles corresponded to a common mid-ROM angle amongst all participants. Work (expressed in J) was defined as the integral of torque with respect to the position between V_MAX_ and 90% of the ROM. The muscle-lengthening threshold was defined as the muscle length at the time of EMG onset during a LV stretch. EMG onset during LV stretches were not often present in the LatGas and RecFem [25]. Therefore, this parameter was only calculated for the MedHam and Adds. In all four muscles, muscle-lengthening velocity threshold was defined as the muscle-lengthening velocity at the time of EMG onset during a HV stretch. All muscle lengths and muscle lengthening velocity thresholds were expressed as a percentage of the muscle length in the anatomical zero position (ML and MLV, expressed as % and %/s, respectively). The angle of catch (AOC) was defined as the angle that corresponded to the time of the first local minimum power after the time that maximum power was reached [36], and was expressed as a percentage of the ROM. To provide a measure of the severity of spasticity, the absolute change between the average of 3–4 repetitions from HV and LV stretch repetitions (^HV-LV^) were calculated for rms-EMG, Torque and Work.

For the intra-rater^WS^ analysis, only ROM, V_MAX_, ML and MLV were calculated. For the inter-rater^WS^ and intra-rater^BS^ analyses, ROM, V_MAX_, rms-EMG^HV-LV^, Torque^HV-LV^, Work^HV-LV^, ML and MLV were calculated by taking the average of 3–4 good stretch repetitions per velocity. AOC was calculated from the first well performed HV stretch, and its reliability was only evaluated for the inter-rater^WS^ and intra-rater^BS^ analyses. The reliability of MVIC was only evaluated for the intra-rater^BS^ analysis.

### Statistical analysis

Group descriptive statistics of all parameters were calculated per muscle and measurement session. Bland-Altman plots portraying limits of agreement were created and independently reviewed by two raters to determine any systematic bias. Relative and absolute reliability were evaluated using the intra-class correlation coefficients (ICC 2,1 for intra-rater^WS^ and ICC 2,k for inter-rater^WS^ and intra-rater^BS^) with 95% confidence intervals [37] and the standard error of measurement (SEM), respectively. The reliability of the data selection procedure was determined by calculating the ICC (ICC 2,k) and SEM on the data curated by raters one and three. The ICC was investigated for absolute agreement to detect any relevant systematic error between raters. The SEM was calculated from the square root of the mean square error from one-way ANOVA, and expressed as a percentage of the mean of the test and re-test values [23]. SEM% values <20% were considered acceptable based upon the average change in previously reported ISA parameters following treatment with BTX in the MedHam [25,26]. ICCs >0.80 indicated high relative reliability, 0.60–0.79 indicated moderately-high relative reliability, 0.40–0.59 indicated moderate relative reliability and <0.40 indicated low relative reliability [38]. To identify the most responsive spasticity-related parameters, the minimal detectable change (MDC) was calculated (MDC = SEM x 1.645 x √2) [39], and expressed as a percentage of the mean of the test and re-test values. Statistical analysis was performed using MATLAB 7.6.0 R2013a (MathWorks), SPSS Statistics (version 22 IBM), and MedCalc (version 12.7).

## Results

Twelve children participated in the study (Table 1). One child participated only in the inter-rater^WS^ analysis, and two children participated only in the intra-rater^WS&BS^ analysis. This yielded a total of 11 children for the intra-rater^WS&BS^ analyses, and 10 children for the inter-rater^WS^ analysis. Data of two RecFem and one Adds were excluded due to time restrictions at the time of data collection, or due to poor quality EMG. The ML parameter was not calculated for two MedHam and five Adds in the intra-rater^WS&BS^ analyses, and for one MedHam and four Adds in the inter-rater^WS^ analysis, due to a lack of EMG onset at LV. Similarly, due to a lack of EMG onset at HV, the MLV parameter was not calculated for two MedHam and two Adds in the intra-rater^WS&BS^ analyses, and for one MedHam and one Adds in the inter-rater^WS^ analysis.

**Table 1:** Patient Characteristics.

Characteristics		Participants (n = 12)							
Mean age (SD)		12.8 years (4.13 years)							
Gender		8 Males; 4 Females							
Clinical Diagnosis		5 Unilateral CP (3 RH; 2 LH) 7 Bilateral CP (6 Di; 1 Quad)							
GMFCS level (I–IV)		level I (n = 6); level II (n = 5); level IV (n = 1)							
Participants per analysis		Intra^WS&BS^	Inter^WS^	Intra^WS&BS^	Inter^WS^	Intra^WS&BS^	Inter^WS^	Inter^WS&BS^	Inter^WS^
		LatGas	LatGas	MedHam	MedHam	RecFem	RecFem	Adds	Adds
		(n = 11)	(n = 10)	(n = 11)	(n = 10)	(n = 9)	(n = 9)	(n = 9)	(n = 9)
MAS score (n = 12)	*0*	0		0		4		1	
	*1*	0		0		2		5	
	*1+*	5		3		3		2	
	*2*	5		8		2		3	
	*3*	2		1		1		0	
Average MTS ° (SD)		LatGas (n = 12)		MedHam (n = 12)		RecFem		Adds	
		7.91° (12.14°)		80.33° (6.49°)		NA		NA	

CP, cerebral palsy; RH, right hemiplegia; LH, left hemiplegia; Di, diplegia; Quad, quadriplegia; GMFCS, Gross Motor Function Classification Score; Intra^WS^, intra-rater within session; Intra^BS^, intra-rater between session; Inter^WS^, inter-rater within session; LatGas, lateral gastrocnemius; MedHam, medial hamstrings; RecFem, rectus femoris; Adds, hip adductors; MAS, Modified Ashworth Scale; MTS, R2 of the Modified Tardieu Score; NA, not applicable.

### Data selection

Following the selection of the 1249 stretch repetitions from the inter-rater^WS^ and intra-rater^BS^ analyses, 139 (11%) were excluded. From the session curated by raters one and three (total 570 stretch repetitions), rater one excluded 131 stretch repetitions (23%) and rater three excluded 76 stretch repetitions (13%). Table 2 reports the subsequent ICC and SEM% values of the data curated by the two raters. Of all the 39 ICC values, two (MLV in the LatGas and AOC in the RecFem) were <0.6. The ICC of the ML for the Adds was not computable. This happens when the between-subject variation is relatively small compared to the within-subject variation.

**Table 2:** Intra-class correlation coefficients (ICC) and the standard error of measurement (SEM%) for the data curated by two raters.

Parameters	LatGas		MedHam		RecFem		Adds	
	ICC	SEM%	ICC	SEM%	ICC	SEM%	ICC	SEM%
V_MAX_^LV^ (°/s)	0.98	4.72	1.00	0.95	0.95	4.29	0.76	23.06
V_MAX_^HV^ (°/s)	0.89	3.37	0.98	2.81	0.94	2.01	0.87	15.25
ROM^LV^ (°)	0.99	2.14	1.00	0.10	0.89	1.20	0.73	13.62
ROM^HV^ (°)	0.95	4.86	1.00	0.55	0.92	0.96	0.94	8.41
rms-EMG ^HV-LV^ (%)	0.98	14.88	0.99	3.90	0.96	4.46	0.94	22.30
rms-EMG ^HV-LV^ (mV)	0.98	20.00	0.99	7.14	0.97	5.00	0.94	22.45
Torque ^HV-LV^ (Nm)	0.99	9.46	0.72	31.08	0.96	3.56	NA	NA
Work ^HV-LV^ (J)	0.99	4.96	0.99	4.52	0.97	4.11	NA	NA
AOC (%)	0.77	6.43	0.98	17.28	0.23	19.25	NA	NA
ML (%)	NA	NA	0.99	0.81	NA	NA	A	4.76
MLV (%/s)	0.34	25.36	0.99	3.96	0.99	0.78	0.67	38.09

Inter^BS^, inter-rater between session; LatGas, lateral gastrocnemius; MedHam, medial hamstrings; RecFem, rectus femoris; Adds, hip adductors; NA, not applicable. A = an ICC that could not be calculated.

SEM% values <20% were found in all but one of the 16 performance-related parameters, the exception being V_max_^LV^ for the Adds. For the spasticity-related parameters, SEM% values <20% were found in all but five of the 23 parameters (MLV in the LatGas and Adds, Torque of MedHam, and rms-EMG and rms-EMG % of the Adds).

### The intra-rater^WS^, inter-rater^WS^, and intra-rater^BS^ analyses

Results from the reliability analyses for the LatGas and MedHam can be found in Table 3, and those for the RecFem and Adds in Table 4. Parameters computed using HV-LV, tended to have higher SD values. This was especially the case for the rms-EMG^HV-LV^ parameters. There was no evidence of systematic bias or heteroscedasticity.

**Table 3:** Averages and SD for parameters of LatGas and MedHam in all sessions, and ICC, CI, SEM and MDC for intra- and inter-rater reliability.

Muscle / Parameters	Test-Retest Mean (SD)							ICC			SEM (SEM%)			MDC (MDC%)		
LatGas	Intra^WS^ rep. 1	Intra^WS^ rep. 2	Intra^WS^ rep. 3	Inter^WS^ rater 1	Inter^WS^ rater 2	Intra^BS^ session 1	Intra^BS^ session 2	Intra^WS^	Inter^WS^	Intra^BS^	Intra^WS^	Inter^WS^	Intra^BS^	Intra^WS^	Inter^WS^	Intra^BS^
V_MAX_^LV^ (°/s)	14.9 (±4.01)	15.95 (±6.83)	18.05 (±6.30)	12.38 (±3.09)	11.75 (±3.23)	16.48 (±5.40)	14.92 (±7.37)	0.87	0.88	0.92	2.96 (18.16)	1.41 (11.70)	2.31 (14.74)	6.86 (42.03)	3.27 (27.09)	5.37 (34.18)
V_MAX_^HV^ (°/s)	154.12 (±33.00)	165.99 (±32.12)	165.49 (±31.82)	141.05 (±7.95)	149.58 (±22.55)	163.10 (±29.41)	161.84 (±21.36)	0.92	A	0.84	13.15 (8.12)	19.56 (13.45)	13.66 (8.41)	30.49 (18.83)	45.36 (31.21)	31.69 (19.50)
ROM^LV^ (°)	51.27 (±9.08)	49.94 (±8.80)	50.63 (±9.11)	49.94 (±8.03)	49.77 (±11.00)	50.71 (±8.82)	49.69 (±6.62)	0.98	0.83	0.86	1.86 (3.68)	5.32 (10.67)	3.89 (7.76)	4.31 (8.51)	12.34 (24.74)	9.04 (18.00)
ROM^HV^ (°)	47.86 (±8.51)	48.32 (±8.38)	48.84 (±8.75)	46.83 (±6.96)	46.99 (±8.38)	48.67 (±8.33)	47.74 (±5.73)	0.97	0.95	0.90	2.38 (4.93)	2.24 (4.79)	2.98 (6.19)	5.51 (11.39)	5.21 (11.10)	6.92 (14.34)
MVIC (mV)	NA	NA	NA	NA	NA	0.08 (±0.004)	0.08 (±0.05)	NA	NA	0.89	NA	NA	0.02 (26.06)	NA	NA	0.05 (57.64)
rms-EMG ^HV-LV^ (%)	NA	NA	NA	6.41 (±7.26)	8.77 (±4.78)	5.69 (±4.78)	6.82 (±7.59)	NA	0.77	0.41	NA	3.53 (46.54)	5.56 (88.88)	NA	8.19 (107.83)	12.90 (206.05)
rms-EMG ^HV-LV^ (mV)	NA	NA	NA	0.004 (±0.003)	0.006 (±0.004)	0.004 (±0.003)	0.003 (±0.002)	NA	0.81	0.81	NA	0.001 (33.71)	0.001 (47.60)	NA	0.002 (38.24)	0.002 (50.00)
Torque ^HV-LV^ (Nm)	NA	NA	NA	5.89 (±4.40)	5.73 (±3.54)	5.80 (±5.00)	5.39 (±4.39)	NA	0.69	0.94	NA	2.82 (48.60)	1.55 (27.81)	NA	6.55 (112.70)	3.61 (64.49)
Work ^HV-LV^ (J)	NA	NA	NA	3.13 (±1.53)	2.91 (±0.93)	3.13 (±1.46)	3.12 (±1.31)	NA	0.68	0.95	NA	0.89 (29.60)	0.4 (13.01)	NA	2.07 (68.38)	0.94 (30.04)
AOC (%)	NA	NA	NA	63.98 (±7.12)	65.25 (±11.34)	70.79 (±7.62)	70.69 (±10.02)	NA	0.40	0.85	NA	8.41 (13.02)	4.72 (6.67)	NA	19.52 (30.20)	10.95 (15.47)
MLV (%/s)	25.96 (±6.41)	26.39 (±7.58)	28.23 (±3.03)	20.59 (±8.52)	24.99 (±4.75)	27.76 (±5.77)	27.36 (±4.35)	0.88	0.47	0.94	3.57 (13.29)	6.87 (30.15)	2.9 (10.53)	8.27 (30.79)	15.94 (69.92)	6.73 (24.41)
**MedHam**																
V_MAX_^LV^ (°/s)	16.76 (±6.22)	18.97 (±10.68)	16.80 (±6.92)	14.66 (±4.62)	12.58 (±3.52)	17.35 (±7.29)	17.68 (±6.03)	0.88	0.58	0.86	4.41 (25.18)	3.01 (22.14)	3.36 (19.20)	10.22 (58.34)	6.99 (51.30)	7.80 (44.52)
V_MAX_^HV^ (°/s)	266.75 (±44.10)	271.49 (±40.81)	267.50 (±46.43)	258.02 (±42.18)	258.9 (±39.66)	270.6 (±41.49)	269.6 (±44.54)	0.95	0.97	0.91	15.12 (5.62)	9.81 (3.79)	17.52 (6.48)	35.06 (13.05)	22.75 (8.80)	40.63 (15.04)
ROM^LV^ (°)	73.69 (±13.09)	72.11 (±15.01)	70.69 (±15.48)	71.82 (±17.25)	68.03 (±15.96)	72.15 (±14.14)	74.44 (14.63)	0.98	0.91	0.96	3.23 (4.47)	6.38 (9.12)	3.49 (4.77)	7.49 (10.37)	14.8 (21.16)	8.11 (11.06)
ROM^HV^ (°)	74.94 (±12.76)	76.10 (±14.63)	76.12 (±15.63)	74.03 (±14.86)	69.51 (±16.69)	75.79 (±14.31)	78.09 (13.79)	0.98	0.88	0.93	2.74 (3.61)	6.94 (9.67)	4.88 (6.35)	6.35 (8.38)	16.09 (22.41)	11.33 (14.72)
MVIC (mV)	NA	NA	NA	NA	NA	0.149 (±0.100)	0.148 (±0.08)	NA	NA	0.90	NA	NA	0.04 (27.03)	NA	NA	0.09 (60.42)
rms-EMG ^HV-LV^ (%)	NA	NA	NA	10.18 (±4.50)	11.87 (±6.00)	11.83 (±6.71)	11.74 (±4.97)	NA	0.49	0.82	NA	4.38 (39.73)	3.33 (28.03)	NA	10.16 (92.12)	7.73 (65.57)
rms-EMG ^HV-LV^ (mV)	NA	NA	NA	0.013 (±0.012)	0.015 (±0.015)	0.015 (±0.011)	0.015 (±0.011)	NA	0.95	0.97	NA	0.004 (28.07)	0.002 (16.27)	NA	0.009 (60.99)	0.005 (31.96)
Torque ^HV-LV^ (Nm)	NA	NA	NA	9.49 (±4.38)	9.75 (±4.89)	8.43 (±4.82)	9.36 (±4.64)	NA	0.45	0.93	NA	4.03 (41.90)	1.64 (10.48)	NA	9.35 (97.12)	3.81 (42.82)
Work ^HV-LV^ (J)	NA	NA	NA	6.89 (±3.44)	5.94 (±3.03)	6.14 (±3.62)	6.32 (±3.40)	NA	0.83	0.92	NA	1.7 (26.50)	1.35 (21.68)	NA	3.94 (61.38)	3.13 (50.17)
AOC (%)	NA	NA	NA	76.37 (±12.84)	79.14 (±17.18)	79.57 (±14.23)	78.41 (±10.31)	NA	0.91	0.92	NA	7.2 (9.26)	4.9 (6.20)	NA	16.71 (21.48)	11.36 (14.38)
ML (%)	88.05 (±2.56)	87.40 (±2.58)	87.43 (±2.56)	87.68 (±2.73)	87.43 (±2.39)	87.36 (±2.45)	87.82 (±2.18)	0.96	0.99	1	0.81 (0.92)	1.36 (1.56)	0.75 (0.86)	1.87 (2.13)	3.16 (3.60)	1.76 (2.00)
MLV (%/s)	26.58 (±12.78)	28.70 (±13.36)	27.34 (11.11)	26.08 (±7.55)	26.40 (±10.18)	27.51 (±11.91)	29.37 (±8.86)	0.97	0.96	0.85	3.14 (11.43)	2.89 (11.04)	5.96 (20.97)	7.28 (26.42)	6.71 (25.57)	13.83 (48.61)

Intra^WS^, intra-rater within session; Intra^BS^, intra-rater between session; Inter^WS^, inter-rater within session; rep, repetition; ^LV^, Low Velocity; ^HV^, High Velocity; ^HV-LV^, Difference between ^HV^ and ^LV^; V_MAX_, Maximum Velocity; ROM, Range of Motion; MVIC, Maximum Voluntary Isometric Contraction; rms-EMG, root mean squared electromyography; AOC, Angle of Catch; ML, Muscle Length; MLV, Muscle Lengthening Velocity. NA = Not Applicable; A = an ICC that could not be calculated. Performance-related parameters appear above the dashed line. Spasticity-related parameters appear below the dashed line.

**Table 4:** Averages and SD for parameters of RecFem and Adds in all sessions, and ICC, CI, SEM and MDC for intra- and inter-rater reliability.

Muscle / Parameters	Test-Retest Mean (SD)							ICC			SEM (SEM%)			MDC (MDC%)		
RecFem	Intra^WS^ rep. 1	Intra^WS^ rep. 2	Intra^WS^ rep. 3	Inter^WS^ rater 1	Inter^WS^ rater 2	Intra^BS^ session 1	Intra^BS^ session 2	Intra^WS^	Inter^WS^	Intra^BS^	Intra^WS^	Inter^WS^	Intra^BS^	Intra^WS^	Inter^WS^	Intra^BS^
V_max_^LV^ (°/s)	18.77 (±5.43)	19.48 (±7.82)	18.75 (±4.16)	15.57 (±2.41)	15.62 (±3.87)	19.09 (±5.64)	19.42 (±7.34)	0.94	0.40	0.89	2.40 (12.64)	2.89 (18.53)	2.95 (15.34)	5.56 (29.25)	6.70 (42.94)	6.85 (35.56)
V_max_^HV^ (°/s)	265.7 (±45.81)	247.78 (±31.19)	252.72 (±31.71)	238.85 (±43.99)	242.13 (±52.37)	254.9 (±32.19)	244.42 (±28.54)	0.84	0.94	0.54	21.52 (8.42)	16.42 (6.83)	24.13 (9.66)	49.91 (19.53)	38.09 (15.83)	55.97 (22.41)
ROM^LV^ (°)	88.85 (±11.59)	86.73 (±10.00)	88.74 (±9.69)	91.34 (±15.28)	90.17 (±14.91)	88.61 (±10.31)	90.41 (±16.06)	0.96	0.91	0.90	3.21 (3.63)	6.21 (6.84)	5.68 (6.34)	7.44 (8.44)	14.41 (15.87)	13.18 (14.72)
ROM^HV^ (°)	88.51 (±8.93)	90.37 (±9.90)	90.01 (±8.61)	93.24 (±13.51)	92.88 (±11.74)	89.73 (±8.53)	91.9 (±10.30)	0.96	0.93	0.73	2.65 (2.96)	4.64 (4.99)	6.23 (6.86)	6.14 (6.85)	10.77 (11.57)	14.46 (15.92)
MVIC (mV)	NA	NA	NA	NA	NA	0.142 (±0.09)	0.147 (±0.08)	NA	NA	0.63	NA	NA	0.06 (46.99)	NA	NA	0.15 (103.52)
rms-EMG ^HV-LV^ (%)	NA	NA	NA	21.27 (±20.50)	17.7 (±16.44)	19.91 (±15.05)	23.07 (±19.47)	NA	0.97	0.86	NA	3.27 (16.81)	8.79 (40.93)	NA	7.60 (38.99)	20.40 (94.91)
rms-EMG ^HV-LV^ (mV)	NA	NA	NA	0.02 (±0.033)	0.02 (±0.027)	0.027 (±0.033)	0.027 (±0.031)	NA	0.98	0.98	NA	0.004 (18.57)	0.005 (18.65)	NA	0.01 (37.63)	0.01 (36.58)
Torque ^HV-LV^ (Nm)	NA	NA	NA	13.19 (±8.90)	12.03 (±7.44)	12.84 (±9.77)	12.92 (±9.26)	NA	0.96	0.98	NA	2.22 (17.66)	1.74 (13.54)	NA	5.16 (40.90)	4.04 (31.34)
Work ^HV-LV^ (J)	NA	NA	NA	9.73 (±5.15)	8.74 (±4.79)	9.09 (±6.93)	8.91 (±5.72)	NA	0.97	0.95	NA	1.04 (11.27)	1.90 (21.17)	NA	2.41 (26.08)	4.42 (49.09)
AOC (%)	NA	NA	NA	74.10 (±16.11)	76.93 (±20.14)	77.66 (±16.32)	78.87 (±15.71)	NA	0.64	0.86	NA	13.73 (18.18)	8.23 (10.51)	NA	31.84 (42.16)	19.09 (24.39)
MLV (%/s)	43.24 (±6.80)	40.63 (±6.27)	38.64 (±6.66)	41.53 (±8.41)	37.9 (±7.36)	40.64 (±5.74)	40.06 (±4.83)	0.80	0.95	0.94	3.98 (9.75)	2.81 (7.07)	4.19 (10.39)	9.23 (22.59)	6.52 (16.41)	9.72 (24.08)
**Adds**																
V_max_^LV^ (°/s)	11.05 (±3.20)	11.01 (±2.56)	11.71 (±2.88)	11.24 (±3.67)	10.78 (±3.61)	11.27 (±2.71)	11.51 (±4.02)	0.85	0.61	0.28	1.72 (15.32)	2.80 (25.50)	3.22 (28.26)	3.98 (35.34)	6.51 (59.09)	7.47 (65.53)
V_max_^HV^ (°/s)	93.09 (±21.88)	105.19 (±25.15)	99.51 (±27.71)	96.48 (±28.35)	84.36 (±21.33)	98.72 (±22.20)	83.77 (±22.25)	0.90	0.69	0.74	11.52 (11.61)	16.03 (17.73)	10.96 (12.01)	26.71 (26.90)	37.19 (41.12)	25.42 (27.85)
ROM^LV^ (°)	34.63 (±10.66)	35.31 (±10.91)	37.01 (±10.09)	35.89 (±7.78)	32.54 (±6.59)	36.16 (±11.12)	33.36 (±6.16)	0.96	0.68	0.47	3.31 (9.28)	4.72 (13.80)	7.53 (21.66)	7.67 (21.51)	10.95 (31.99)	17.47 (50.25)
ROM^HV^ (°)	33.06 (±6.99)	36.00 (±7.85)	35.40 (±10.35)	33.86 (±7.58)	30.76 (±8.72)	34.77 (±8.18)	30.66 (±7.57)	0.93	0.82	0.77	3.24 (9.31)	4.19 (12.99)	4.21 (12.87)	7.51 (21.56)	9.73 (30.11)	9.76 (29.82)
MVIC (mV)	NA	NA	NA	NA	NA	0.096 (±0.063)	0.104 (±0.083)	NA	NA	0.91	NA	NA	0.02 (29.81)	NA	NA	0.06 (59.83)
rms-EMG ^HV-LV^ (%)	NA	NA	NA	8.61 (±4.63)	7.89 (±6.74)	9.02 (±4.99)	7.05 (±5.57)	NA	0.79	0.78	NA	3.51 (42.65)	2.99 (37.22)	NA	8.16 (98.87)	6.94 (86.30)
rms-EMG ^HV-LV^ (mV)	NA	NA	NA	0.007 (±0.004)	0.006 (±0.005)	0.007 (±0.004)	0.005 (±0.004)	NA	0.81	0.86	NA	0.003 (44.90)	0.002 (32.77)	NA	0.007 (102.02)	0.004(63.39)
ML (%)	107.79 (±7.42)	108.01 (±5.40)	107.08 (±7.51)	103.66 (±4.22)	109.88 (±5.93)	107.93 (±6.31)	106.5 (±4.91)	0.91	0.98	0.99	3.30 (3.07)	5.40 (5.05)	1.60 (1.49)	7.65 (7.10)	12.53 (11.73)	3.72 (3.46)
MLV (%/s)	47.53 (±27.71)	51.00 (±28.49)	46.09 (±30.65)	33.19 (±16.89)	32.65 (±16.70)	38.15 (±14.57)	32.22 (±12.86)	0.97	0.86	0.68	7.28 (15.11)	8.92 (27.09)	10.55 (29.98)	16.88 (35.24)	20.68 (62.81)	24.47 (69.53)

Intra^WS^, intra-rater within session; Intra^BS^, intra-rater between session; Inter^WS^, inter-rater within session; rep, repetition; ^LV^, Low Velocity; ^HV^, High Velocity; ^HV-LV^, Difference between ^HV^ and ^LV^; V_MAX_, Maximum Velocity; ROM, Range of Motion; MVIC, Maximum Voluntary Isometric Contraction; rms-EMG, root mean squared electromyography; AOC, Angle of Catch; ML, Muscle Length; MLV, Muscle Lengthening Velocity. NA = Not Applicable. Performance-related parameters appear above the dashed line. Spasticity-related parameters appear below the dashed line.

Of all the ICC values, 76% were >0.8 and 14% >0.6 (Table 5). Of the 11 ICC values <0.6, four were in the intra-rater^BS^ analysis, and seven in the inter-rater^WS^ analysis. There were three V_max_^LV^; two V_max_^HV^; two rms-EMG^HV-LV^ (%); one ROM^LV^; one Torque^HV-LV^; one AOC and one MLV. Four were found in the LatGas, three in the MedHam, and two in both the RecFem and Adds.

**Table 5:** The number of parameters in all three analyses categorised according to their intra-class correlation coefficient (ICC) and standard error of measurement (SEM) and expressed as a percentage of the mean test and re-test values for all four muscles.

ICC	>0.8	>0.6	>0.4	<0.4	Total	SEM	0–10%	11–20%	21–30%	>30%	Total
**LatGas**	19	4	3	1	*27*	**LatGas**	8	10	3	6	*27*
**MedHam**	28	0	3	0	*31*	**MedHam**	16	4	8	2	*30*
**RecFem**	22	3	2	0	*27*	**RecFem**	11	13	1	2	*27*
**Adds**	12	8	1	1	*22*	**Adds**	5	8	6	4	*23*
**Total**	*81*	*15*	*9*	*2*	*107*	**Total**	*40*	*35*	*18*	*14*	*107*
**% of total**	**76**	**14**	**8**	**2**	**100**	**% of total**	**37**	**33**	**17**	**13**	**100**
**Total % with ICC >0.6**	**90%**					**Total % with SEM <20%**	**70%**				

LatGas, lateral gastrocnemius; MedHam, medial hamstrings; RecFem, rectus femoris; Adds, hip adductors.

**Fig 4:**
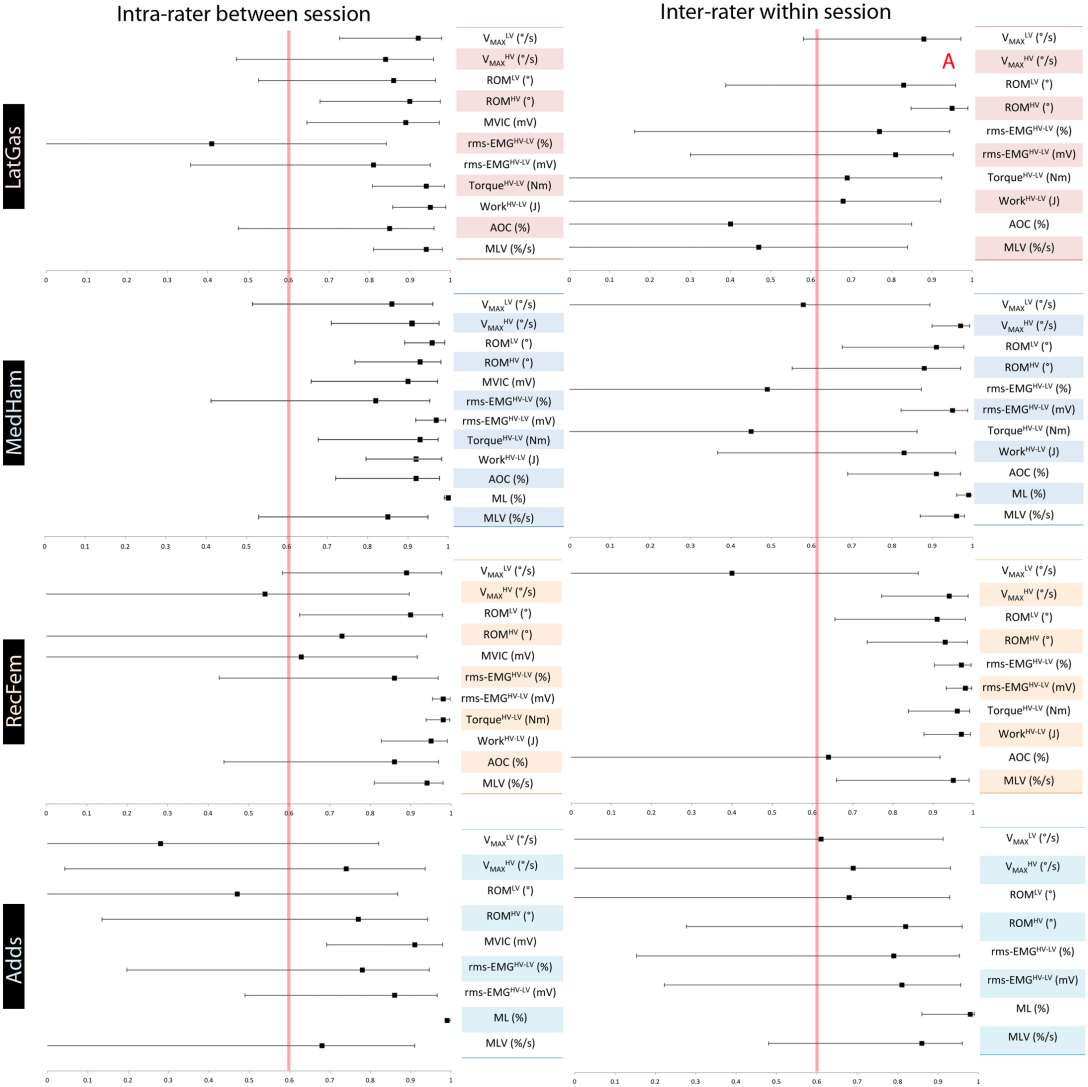
The intra-class correlation coefficients (ICC) and confidence intervals (CI) for intra-rater^BS^ and inter-rater^WS^ analyses. LatGas, lateral gastrocnemius; MedHam, medial hamstrings; RecFem, rectus femoris; Adds, hip adductors; LV, Low Velocity; HV, High Velocity; HV-LV, Difference between HV and LV; V_MAX_, Maximum angular velocity; ROM, Range of Motion; MVIC, Maximum Voluntary Isometric Contraction; rms-EMG, root mean squared electromyography; AOC, Angle of Catch; ML, Muscle Length; MLV, Muscle Lengthening Velocity. The red vertical line indicates an ICC of 0.6, above which relative reliability is considered to be at least moderately high. A = an ICC that could not be calculated.

ICC values with their corresponding confidence intervals for inter-rater^WS^ and intra-rater^BS^ are displayed in Fig 4. In the LatGas and MedHam, overall wider CIs of the ICC values were seen for the inter-rater^WS^ than for the intra-rater^BS^, except for the rms-EMG^HV-LV^ (%), which was wide in both analyses. With the exception of V_max_^LV^ and AOC, the opposite trend was seen for the RecFem. CIs of both Adds analyses were similar, but generally wider than those in the other muscles.

### Standard error of measurement (SEM)

For the SEM values of all four muscles, expressed as a percentage of the average of the mean of the test and re-test values, 37% were below 10% error, 33% were between 11–20% error, 17% were between 21–30% error and 13% were ≥30% error (Table 5). Of those 32 SEM values >20%, 17 were found in the intra-rater^BS^ analysis, 14 were found in the inter-rater^WS^ analysis and one in the intra-rater^WS^ analysis. The higher SEM values were seven rms-EMG^HV-LV^ (%); five rms-EMG^HV-LV^ (mV); four V_max_^LV^; four Work^HV-LV^; four MVIC; four MLV; three Torque^HV-LV^; and one ROM^LV^, and were more often found in the RecFem and Adds than in the LatGas and MedHam.

## Discussion

This study evaluated the reliability of an instrumented assessment tool integrating multidimensional signals in order to quantify spasticity in children with spastic CP. The different sources of intrinsic and extrinsic errors associated with ISA were comprehensively analysed in this study. ISA was found to be reliable in all of the three reliability analyses, with 90% of the parameters showing ICC values >0.6, and 70% of the SEM% values <20%. In most cases, ICC values >0.6 were accompanied by SEM% values <20%. This confirmed our first hypothesis that parameters investigated with ISA are overall reliable.

### Reliability

#### Intra-rater^WS^ analysis

The intra-rater^WS^ analysis compared the first three good quality stretch repetitions in the same measurement session. This assessed for any error inherent to the investigated parameters. Such error may be caused by intrinsic factors such as spasticity, post activation depression, thixotropy, or an extrinsic error like the waiting time between stretch repetitions. In this analysis, most parameters showed an ICC >0.8 and SEM% values <20%. SEM% values were comparable to, if not smaller than the values from the two other reliability analyses. This finding confirms a limited contribution of error due to three repeated stretch repetitions, and infers that a seven second waiting period is satisfactory, allowing for the influence of any hyper-excitability or post activation depression of a muscle stretch to subside [25].

#### Intra-rater^BS^ analysis

After the intra-rater^WS^ analysis, the second most reliable analysis was the intra-rater^BS^, where extrinsic errors introduced between sessions were analysed. Re-application of the IMU sensors in different sessions requires a new calibration procedure, possibly influencing the joint motion parameters. A similar justification can also be made for the re-application of the sEMG electrodes and orthoses, which may influence the spasticity-related parameters and the handling of a stretch. Additionally, the participant and the limb on the support frame need to be repositioned. Nonetheless, the intra-rater^BS^ analysis still demonstrated a satisfactory level of reliability. In order to further improve a between session analysis, the sources of extrinsic error should be accounted for and reduced. Bar-On et al. have previously evaluated the reliability for the intra-rater^WS&BS^ analyses for several parameters of the LatGas and MedHam [25]. In comparison with the current study, they showed lower ICC and generally higher SEM values for all performance- and some spasticity-related parameters. This finding was expected as their study included only six participants, which may not have been a representative sample. Furthermore, in contrast to the two-hour interval between measurement sessions of the current study, Bar-On et al. reported an average interval of 13 days [25]. Too short an interval may interfere with the participants’ concentration, whilst too long an interval makes it challenging to control what happens during the interim period. The appropriate time interval for a between session reliability analysis should be further investigated.

#### Inter-rater^WS^ analysis

The reliability of ISA was generally higher when comparing within and between sessions performed by the same rater, than between two different raters. Inter-rater reliability is significant if ISA is to be used in clinical practice, as the same rater is not always available to perform a follow up assessment. Furthermore, considering that the current inter-rater analysis investigated within the same session, additional extrinsic errors are also anticipated between sessions. Standardisation and training should be further improved to increase the reliability when different raters perform the measurement. This could be achieved by ensuring that different raters practice together when learning how to grasp the loadcell, where to stand when performing each measurement, the addition of a metronome beep to suggest and support specific stretch velocities, and by the use of training videos.

#### Investigated muscles

When comparing the four muscles, the performance-related parameters had a tendency to be most reliable in the MedHam, followed by LatGas and RecFem, and then Adds. For the spasticity-related parameters, the RecFem had the highest reliability, followed by MedHam and LatGas, and then Adds. It is not so surprising that the Adds were the least reliable of the investigated muscles, as they are also the most difficult stretch to perform. It requires movement of the entire limb, as opposed to just a single segment, which may allow a larger introduction of errors. Furthermore, identifying only one of the adductor muscles is challenging in children with CP, and crosstalk between muscles may have occurred. Additionally, the nature of spasticity in the Adds may have a higher intrinsic error than the other three muscles. This could not be confirmed by the current study, as indications of spasticity severity (HV-LV) were not computable in the intra-rater^WS^ analysis, and comparisons between different muscles with spasticity have not been reported in literature.

### The implications of data selection

Since ISA is a manually performed test, the selection procedure is essential in ensuring that only well performed stretch repetitions are included for analysis. However, as the selection procedure was not automated, it has to be considered as a possible source of extrinsic error. Two raters independently curated the same set of data, following the same rules of data exclusion. The final number of included stretch repetitions varied between the two raters (excluded: rater one = 23%; rater three = 13%). Despite these differences, small SEM% values were found in all but five of the 23 spasticity-related parameters. The exception was the MLV parameter in the LatGas and Adds. This parameter was calculated by defining the timing of EMG onset. In those cases when neither automatic EMG onset detection method was successful, the EMG onset was manually determined, which may explain some of the discrepancy between raters. Another exception was the Torque parameter of MedHam. Stretch repetitions were seldom excluded due to artefacts in the torque signal. Therefore, exclusion of stretch repetitions based on other criteria was the likely cause of a high SEM% for the torque parameter. Lastly, low selection agreement between raters also influenced the two rms-EMG parameters of the Adds. This may have been caused by the high EMG baseline often seen in the Adds. Overall though, the investigation of the data selection procedure confirmed the hypothesis that little extrinsic error is introduced, as long as three well-performed stretch repetitions are available, and that both raters adhere to the well-defined selection criteria. In the future, the addition of a live feedback system informing the clinician in real time about each stretch repetition, will avoid the issue of capturing excess data to provide at least three well-performed stretch repetitions.

### ISA compared to other literature

To the best of the author’s knowledge, only six other groups evaluated the reliability of a manually controlled device that combines multidimensional signals for the assessment of spasticity (Table 6).

**Table 6:** The previously reported manually applied instrumentation that underwent a reliability analyses.

Author	Pathology	Investigated Signals	Intra-rater Analysis	Inter-rater Analysis	Statistical Analysis
Lamontagne (1998) [40]	SCI (n = 9)	Biomechanical	Within session analysis (1 second)	Not performed	ICC / CV
Pandyan (2001) [29]	Stroke (n = 16)	Biomechanical	Within session analysis (10–15 seconds)	Not performed	ANOVA
Van Der Salm (2005) [28]	SCI (n = 9)	Biomechanical	Within session analysis (5 seconds)	Not performed	ICC / CIs
Voerman (2007) [41]	Stroke / Healthy(n = 12/11)	Biomechanical Electrophysiological	Between session analysis (10 minutes)	Between session analysis (1 day)	ICC
Turk (2008) [42]	Stroke / Healthy(n = 12/12)	Biomechanical Electrophysiological	Between session analysis (unspecified time)	Between session analysis (no waiting time)	ICC / SRD / LOA
Wu (2010) [21]	TD (n = 10)	Biomechanical	Between session analysis (1 day)	Not performed	ICC

SCI, Spinal Cord Injury; TD, Typically Developing; ICC, intra-class correlation coefficient; CV, coefficient of variation; CIs, confidence intervals; ANOVA, analysis of variance; SRD, smallest real difference; LOA, limits of agreement.

Overall, the parameters that could be compared to previous studies were shown to be of either similar, or higher reliability in ISA. Although all the studies in Table 6 assessed spasticity with multidimensional signals, only two studies investigated the reliability of both the biomechanical and electrophysiological parameters, and that was in the pathology of stroke [41,42]. Furthermore, no study assessed the reliability of a manually controlled device in CP. For the studies that assessed an intra-rater^WS^ analysis, waiting time between stretch repetitions varied from one second to 15 seconds, suggesting that the seven second time interval selected for ISA is a fair compromise. Between sessions analyses intervals ranged from 10 minutes, to one day, illustrating the obscurity of what is sufficient. Finally, the extent of statistical analyses for assessing reliability varied between studies, and it can be viewed as a limitation that only one study investigated a measure of absolute reliability.

### Implications of findings

Reliability is considered to be the basic psychometric criterion for assessment tools, and without it, validity and responsiveness cannot be determined. The SEM infers that the smaller its value, the fewer the errors (random and systematic), and in turn the greater the reliability [43]. An SEM% value may also be referenced in terms of the responsiveness to treatment. If an SEM value is able to yield an MDC value small enough to detect change post treatment, it can be statistically interpreted as reliable. Based on the results of the current study, we can attempt to assess the clinical feasibility of ISA in its current state. As previously identified, all four investigated muscles had EMG onsets at high velocity, suggesting some component of velocity-dependent spasticity. In addition, the MedHam and Adds also had an EMG onset at low velocity, suggesting a component of position-dependent spasticity. This already suggests a possible distinction for evaluating various types of spastic behaviour. Certain ISA parameters have been deemed sensitive enough to differentiate between pre and post treatment intervention with BTX in the MedHam [26]. In order to validate this finding, the corresponding MDC values of the same spasticity-related parameters from the current study can be compared to the average treatment induced change values reported in literature (Table 7).

**Table 7:** MDC for the spasticity-related parameters of the medial hamstrings (MedHam), and the average difference of those parameters between pre and post treatment with Botulinum Toxin-A (BTX) as previously reported [26].

	MDC Intra-rater^BS^	MDC Inter-rater^WS^	Pre-post BTX [26]
**Spasticity-related parameters**			
rms-EMG^HV-LV^ (mV)	0.005	0.009	**0.009**
Torque^HV-LV^ (Nm)	3.81	9.35	2.82
Work^HV-LV^ (J)	3.13	3.94	1.08
AOC (%)	11.36	16.71	**13.83**

Intra^BS^, intra-rater between session; Inter^WS^, inter-rater within session; rms-EMG, root mean squared electromyography; HV-LV, difference between stretches at high velocity and stretches at low velocity; AOC, Angle of Catch. Numbers in **bold** reflect values larger than their corresponding intra- or inter-rater MDC.

The MDC value of the rms-EMG^HV-LV^ (mV) parameter was small enough to detect a response in the MedHam to treatment with BTX. This is expected because the rms-EMG parameter most closely reflects the definition of spasticity [4]. However, the effect of BTX treatment on the MedHam did not exceed the reported MDC values for the torque and work parameters. These parameters not only reflect spasticity, but also non-neural tissue changes such as increased passive muscle stiffness and viscosity. These non-neural components could account for the parameters’ limited response in detecting a change post BTX [44]. Another consideration is that these parameters are highly dependent on the way the stretch is performed (grasp of the force/torque load-cell). Further research is required to study the effect of tone reduction treatment for all lower limb muscles, using the MDC values of the spasticity related parameters reported by the current study. Additionally, progress is also required to decompose the biomechanical parameters into their neural and non-neural components.

For a device like ISA, the MDC alone is not enough, and it is also important to acknowledge the minimally important change (MIC). The MIC can be established by evaluating the effect of decreasing spasticity on the development of secondary muscle deformities. On a future consideration, changes in function by means of 3D motion analysis, and patient/clinician feedback can also be used.

### Study limitations

Several study limitations need to be acknowledged. The number of participants was small, especially for a reliability study applying parametric statistics. Twelve participants are comparable to the sizes recruited in other studies [21,28,29,40–42], but are still limited taking into account the power analysis estimated by Walter et al [45]. The medium velocity stretch repetitions were excluded from this investigation, as manually acquiring them with ISA is more challenging and time consuming than with a motorized system. In those cases where a low ICC value was combined with a relatively low SEM% value, it can be argued that the ICC may not have been a suitable statistic. The ICC is indicative of relative reliability, so if the sample group is homogenous, ICC values will be small, even if the test-retest variability is small, and vice versa [23]. This limitation necessitated the inclusion of a measure of absolute reliability. If an SEM is high, consideration of the various sources of error can help to determine if it can be reduced [24]. In the case of a high ICC value with a high SEM, this may indicate systematic error. One way to estimate the presence of systematic error over random error is to compare various ICC calculation models [23].

Parameters involving HV-LV calculations often showed poorer reliability. As these parameters were not assessed in the intra-rater^WS^ analysis, further investigation is required to determine where the error is coming from, and if it can be reduced. The MVIC may be difficult to collect in children with CP [46], therefore, it was decided that both normalised and non-normalised rms-EMG parameters would be investigated. Overall, the non-normalised rms-EMG parameter appeared to be more reliable, indicating that the MVIC introduced error. This should be considered in future studies when attempting to detect severity of spasticity or responsiveness to an intervention.

For reasons of feasibility, this study was unable to evaluate the reliability of an inter-rater^BS^ analysis. Based on the findings of the intra-rater^BS^ and inter-rater^WS^ analyses, it is assumed that there will be some degree of error within the parameters of an inter-rater^BS^ analysis. Consequently, without this analysis, if two different raters perform the pre and post measurements of an intervention, it is unknown if the investigated parameters will be sensitive enough to detect a change. This gap remains a limitation in ascertaining the true reliability of ISA in the clinical setting.

As angles were only calculated in the sagittal plane, it was assumed that calibration and stretch trials were only performed within this plane, and in addition, that only one joint was moved during stretch. A previous study reported limited measurement error when small out-of-plane-movements, or movement of the proximal joint occur [25]. Nevertheless, in the current study, participants lacking neutral joint-alignment were excluded, and out-of-plane movements were minimized by means of standardised reporting on the performance of each stretch.

Lastly, inertial influences on torque were estimated with anthropometric approximations, whereby the foot and lower leg were considered as one segment (see appendix 1) [34]. Fortunately, a previous study has shown that the error introduced by assuming the ankle as fixed during knee movements only has a limited effect on the resulting knee-joint torque [25].

## Conclusion

Based on the outcomes of this reliability study, together with the previously published literature, ISA has been demonstrated to possess a wide range of applications in both the research and clinical environment. The sources of error identified within this study seem to be small, and to not have a large impact on the parameters. The intra-rater^WS^ was the most reliable of the three analyses, followed by the intra-rater^BS^, and then the inter-rater^WS^. The time interval between sessions, re-application of sensors and repositioning of the participant are likely sources of error. When two different raters perform the measurement, standardisation and training should be improved to minimise the extrinsic error as much as possible. Errors were also muscle specific, or related to the measurement set-up. This variation needs to be accounted for, especially when assessing pre-post interventions or longitudinal follow-up.

## Supporting Information

S1 Fig Internal Joint Torque Calculations. (TIF)

S2 Fig Measurement procedure for four lower limb muscles. ADDs, adductors; MEHs, medial hamstrings; REF, rectus femoris; GAS, gastrocnemius. The arrow indicates the direction of joint movement during stretch. Instrumentation: (1) two inertial measurement units (joint angle measurement); (2) surface electromyography (muscle activation measurement); and (3) a six DoF force-sensor attached to a shank or foot orthotic (torque measurement); (4) support frame.
(TIF)
