# Supplementary figures and images for: The Intra- and Inter-Rater Reliability of an Instrumented Spasticity Assessment in Children with Cerebral Palsy
Source: PLoS One. 2015 Jul 2;10(7):e0131011. doi: 10.1371/journal.pone.0131011 (PMC4489837; doi:10.1371/journal.pone.0131011)

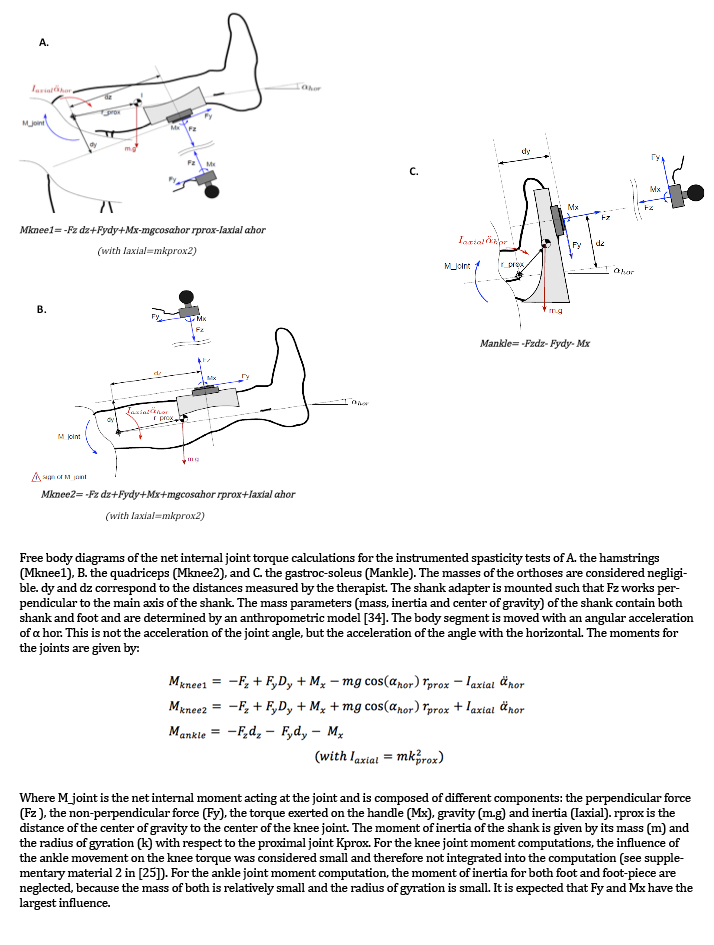

Supplement: S2 Fig — ADDs, adductors; MEHs, medial hamstrings; REF, rectus femoris; GAS, gastrocnemius. The arrow indicates the direction of joint movement during stretch. Instrumentation: (1) two inertial measurement units (joint angle measurement); (2) surface electromyography (muscle activation measurement); and (3) a six DoF force-sensor attached to a shank or foot orthotic (torque measurement); (4) support frame. (TIF) [file pone.0131011.s002.tif]
